# Blood Globules

**Published:** 1883-05

**Authors:** 


BLOOD GLOBULES.

In man the average volume of a red globule is said to be Omm. c. 000,000,072. According to Vievordt and Welcker, a cubic millimeter of blood contains about 5,000,000 red globules. The blood of women contains less. In ten liters of blood the number of globules would be about 50,000 milliards; and the superficies of these globules has been said to be 2,816 square meters. More or less, there is one white globule to 350 or 500 red globules. A cubic millimeter of blood would count about 8,000.—*Wurtz, Chimie Biologique.*

---

THE New York Policlinic has had one hundred and twelve physicians studying in the various classes since November 7th, last, and two thousand five hundred patients have been treated in the Policlinic building within this period.—*Medical News.*

